# Impact of Impure Gas on CO_2_ Capture from Flue Gas Using Carbon Nanotubes: A Molecular Simulation Study

**DOI:** 10.3390/molecules27051627

**Published:** 2022-03-01

**Authors:** Yiru Su, Siyao Liu, Xuechao Gao

**Affiliations:** 1 Key Laboratory of Low-Grade Energy Utilization Technologies and Systems, Ministry of Education, School of Energy and Power Engineering, Chongqing University, Chongqing 400044, China; sophia_yiru@163.com; 2 State Key Laboratory of Pollution Control and Resource Reuse, School of the Environment, Nanjing University, Nanjing 210023, China; 3 State Key Laboratory of Materials-Oriented Chemical Engineering, College of Chemical Engineering, Nanjing Tech University, Nanjing 211816, China

**Keywords:** molecular simulation, gas separation, single-walled carbon nanotube, impure gases

## Abstract

We used a grand canonical Monte Carlo simulation to study the influence of impurities including water vapor, SO_2_, and O_2_ in the flue gas on the adsorption of CO_2_/N_2_ mixture in carbon nanotubes (CNTs) and carboxyl doped CNT arrays. In the presence of single impure gas, SO_2_ yielded the most inhibitions on CO_2_ adsorption, while the influence of water only occurred at low pressure limit (0.1 bar), where a one-dimensional chain of hydrogen-bonded molecules was formed. Further, O_2_ was found to hardly affect the adsorption and separation of CO_2_. With three impurities in flue gas, SO_2_ still played a major role to suppress the adsorption of CO_2_ by reducing the adsorption amount significantly. This was mainly because SO_2_ had a stronger interaction with carbon walls in comparison with CO_2_. The presence of three impurities in flue gas enhanced the adsorption complexity due to the interactions between different species. Modified by hydrophilic carboxyl groups, a large amount of H_2_O occupied the adsorption space outside the tube in the carbon nanotube arrays, and SO_2_ produced competitive adsorption for CO_2_ in the tube. Both of the two effects inhibited the adsorption of CO_2_, but improved the selectivity of CO_2_/N_2_, and the competition between the two determined the adsorption distribution of CO_2_ inside and outside the tube. In addition, it was found that (7, 7) CNT always maintained the best CO_2_/N_2_ adsorption and separation performance in the presence of impurity gas, for both the cases of single CNT and CNT array.

## 1. Introduction

Carbon capture and storage (CCS) [1] technologies have been extensively developed to minimized the influence of CO_2_ emission on the global warming effect. Among the separation techniques, adsorption separation [2] is regarded as a promising solution for its low cost and high efficiency. In this connection, a host of conventional and emerging nanoporous materials have been invented and explored, including zeolites, activated carbons, metal-organic frameworks (MOFs), and carbon nanotubes (CNTs) [3,4,5,6,7,8]. Particularly, CNTs possess large specific surface areas (greater than 1000 m^2^/g) with strong adsorptive affinities, which could be paired with the superior transport properties to further facilitate the adsorption potentials of [3,9,10,11,12,13,14,15,16,17,18,19,20,21,22,23,24] CNTs for CO_2_ capture.

In our previous study, grand canonical Monte Carlo (GCMC) simulations were conducted to investigate the adsorption of CO_2_ in the internal space of individual single CNTs in the presence of pre-adsorbed water [3]. It was found that the pre-loaded water provided additional H_2_O–CO_2_ interactions to facilitate the adsorption of CO_2_, by taking up the adsorption site available for CO_2_. Similarly, as reported by Yu et al. [1], the presence of SO_2_ in the gas phase exerted a negative effect on the adsorption of CO_2_ for CO_2_/N_2_/SO_2_ mixture in HKUST-1 at ambient temperature. By comparison, the presence of O_2_ exerted little effect on the adsorption of CO_2_ in HKUST-1. The main components of flue gases generated by coal-fired power plants include N_2_ (about 73–77%), CO_2_ (15–16%), H_2_O (5–7%), O_2_ (about 3–4%) [12], and trace amounts of SO_2_, etc. [25,26]. Therefore, the impurity gases, such as H_2_O, O_2_, and SO_2_ are expected to impose a significant influence on the adsorption and separation of CO_2_ from flue gas using CNTs [1,3,27,28,29,30].

In practice, oxidation defects often occurred during the acidic/oxidative purification of carbon nanotubes [31], where oxygen-containing functional groups (mainly carbonyl and carboxyl) could be grafted to the defect sites [32]. The oxygen-containing functional groups, such as carboxyl and hydroxyl groups, are hydrophilic, so it could significantly enhance the adsorption of water vapor contained in flue gas, which thus imposes strong influence on the adsorption in CNTs for the rest components in flue gas [33,34]. Further, instead of single carbon nanotube, carbon nanotube bundles were generally obtained during the synthesis procedure. Therefore, to explore the influence of impurity gases on the adsorption and separation of CO_2_ from flue gas in a practical manner, the adsorption of gas mixtures (CO_2_/N_2_/X, X denotes the impurity gases, H_2_O, SO_2_, and O_2_) in the functionalized CNT bundles are required. To the best of our knowledge, the adsorption behavior of impurity gases in the functionalized CNT bundles is still unknown. Hence, the effects of three impurity gases on the separation of CO_2_ in CNT also have not been systematically studied. Different from binary mixture, there are more complex interactions between three impurity gases, the cooperative impact on CO_2_ adsorption has hardly been studied. In addition, little is known about how the cooperative effects between adsorbate-CNT interaction and interaction between impurity and adsorbate affect CO_2_/N_2_ selectivity. Discussions related to these and other related issues will be obtained in detail in this work. Furthermore, the influence on the optimum diameter of CNTs for separating CO_2_/N_2_ is not reported yet.

In this work, GCMC and density functional theory (DFT) simulations were conducted to investigate the adsorption separation of CO_2_ from flue gases using carbon nanotubes in the presence of impurity species (H_2_O, O_2_ and SO_2_), in order to fundamentally reveal the impacts of impurity gases on the adsorption behaviors and separation performance of CO_2_. DFT calculations were specifically conducted to add the carboxyl groups to the vacant oxidation defects of CNTs. Both the adsorption of gas mixtures in single carbon nanotubes and carbon nanotube bundles with functional groups were systematically considered. The separation of SO_2_/N_2_ mixtures also was investigated in CNTs. As both adsorption capacity and selectivity determine the performance of the adsorbents, the performance coefficient of functionalized CNT bundles was used to comprehensively evaluate the CO_2_ separation potential using CNTs.

## 2. Simulation Details

### 2.1. Molecular Models

**Figure 1:**
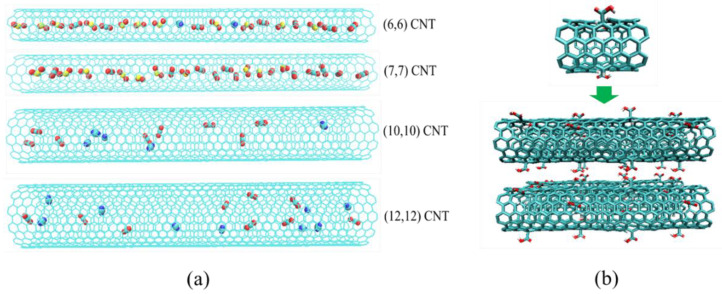
Snapshots of the adsorption of CO_2_/N_2_ mixture in four CNTs in the presence of impurities, at 1.0 bar and 300 K, where the blue and cyan spheres used for N_2_ molecules, while the red and cyan spheres were for CO_2_ molecules, and O_2_ molecules were marked as the red and yellow spheres (e.g., Red = oxygen, yellow = sulfur, cyan = carbon, blue = nitrogen) (**a**). The optimized structure of CNT unit cell with defects, and the constructed 2 × 2 CNT array (**b**).

In our simulations, CO_2_ was modeled by EMP2 model of Harris and Yung [35]. N_2_ and O_2_ were treated as a rigid three-site model with two LJ sites carrying negative charges to represent the N/O atoms, associated with a dummy particle located at center of mass (COM) being used to carry the positive charges to maintain the electrostatic neutralization of molecule [36]. H_2_O was represented by the SPC/E model, which treated H_2_O as a rigid molecule with a positive charges on H atoms and negative partial charges on the O atom [37]. SO_2_ is modeled as a three-site model as well, with a charged LJ particle being assigned for each atom [38]. In addition, the Steele parameters were used to represent the carbon atoms in CNTs. All of the configurational parameters [13], LJ parameters, and partial charges of these guest molecules and the CNTs are summarized in Table 1. The adsorption configuration of gas molecules in four CNTs, the optimized structure of CNT unit cell with defects, and the constructed CNT array can be seen in Figure 1a,b. The interactions of adsorbate–adsorbent and adsorbate–adsorbate are described by the dispersion and electrostatic terms, given by

(1) $$u_{{}_{ij}}^{\left(\alpha ,\beta \right)}=4_{{}_{ij}}^{\left(\alpha ,\beta \right)}\left[{\left(\frac{\sigma_{{}_{ij}}^{\left(\alpha ,\beta \right)}}{r_{{}_{ij}}^{\left(\alpha ,\beta \right)}}\right)}^{12}-{\left(\frac{\sigma_{{}_{ij}}^{\left(\alpha ,\beta \right)}}{r_{{}_{ij}}^{\left(\alpha ,\beta \right)}}\right)}^{6}\right]+\frac{1}{4\pi \varepsilon_{0}}\frac{q_{i}q_{j}}{r_{{}_{ij}}^{\left(\alpha ,\beta \right)}}$$

where $r_{{}_{ij}}^{\left(\alpha ,\beta \right)}$ is the distance between the atom *i* and *j* of molecules α and β. The LJ size parameter $\sigma_{ij}$ and well depth parameter $\varepsilon_{ij}$ for the interactions between different species were estimated using Lorentz–Berthelot mixing rules [39], and the Dot Line Method was used to modify the long range electrostatic interactions in CNTs [40,41].

### 2.2. Grand Canonical Monte Carlo Simulations

To gain insights of the effect of impure gases on the adsorptive separation of CO_2_ from flue gas using CNTs, three impurity gases, SO_2_, H_2_O, and O_2_, were used to conduct simulations for the adsorption in four different CNTs ((6, 6), (7, 7), (10, 10) and (12, 12)), having the diameters of 0.81 to 1.63 nm, were considered. Initially, the adsorption of binary mixture CO_2_/N_2_ in different CNTs was examined to find out the optimized pore size of the CNT for CO_2_ separation. Afterwards, ternary mixtures, including CO_2_/N_2_/SO_2_, CO_2_/N_2_/O_2_, and CO_2_/N_2_/H_2_O were used to determine the effects of individual single impurity on the separation performance of CNTs. Eventually, the simulations for the adsorption of quinary mixture, CO_2_/N_2_/SO_2_/H_2_O/O_2_ was conducted to reveal the effect of the co-existing impure gases on the CO_2_ separation, and the optimal CNT pore size for CO_2_ separation in practice. In all the simulations, the molar ratio of CO_2_/N_2_ mixture was fixed at 16/84 in the bulk phase, while the partial pressure of H_2_O in the ternary mixtures being set as at its saturation pressure of 3.537 kPa, at 300 K. In addition, the mole fraction of SO_2_ and O_2_ in the ternary mixture was set as 0.08% and 4%, respectively. However, for the quinary mixture, the mole fractions of each gas species were defined as: 16 CO_2_: 4 O_2_: 3.16 H_2_O: 0.08 SO_2_ [17], which were chosen to mimic the practical composition of flue gases.

GCMC simulations were conducted to measure the adsorption and separation of CO_2_ from flue gas in consideration of the effects of impurities, the adsorbate chemical potential μ, system volume V, and temperature T were maintained constant during simulations. Three Monte Carlo trial moves including the displacement, insertion, and deletion with corresponding probabilities of 0.4, 0.3, and 0.3 were implemented. The fugacities of the components in the bulk phases were calculated using the Peng–Robinson equation of state [42] (PR EOS) for mixtures. For the binary mixture, 1 × 10^7^ configurations were used to equilibrate the system, which was supplemented by another 5 × 10^7^ configurations for statistical analysis. For the ternary mixtures, the configurations used for equilibration and statistics become 1 × 10^8^ and 2 × 10^8^, respectively. For the quinary mixture, 3 × 10^8^and 6 × 10^8^ configurations were used for equilibration and measuring the isotherm measurement. The equilibrium selectivity, $S_{ij}$, was calculated according to

(2) $$S_{i/j}=\left(\frac{x_{i}}{y_{i}}\right)/\left(\frac{x_{j}}{y_{j}}\right)$$

where, $x_{i}$ and $y_{i}$ were the molar fractions of component *i* in the adsorbed and bulk phases, respectively.

Four kinds of CNTs with different diameters were doped with carboxyl groups to form CNT bundles. Firstly, the original unit cell of carbon nanotube model was established. Carbon atoms were randomly deleted to produce a vacant defect, where each vacant defect contained three sp3 hybrid carbon atoms. The carboxyl group was randomly grafted to one of the SP^3^ hybrid carbon atoms, and hydrogen atoms were added to the other two carbon atoms to saturate the free valence. After the three vacancy defects were modified, the cell was randomly rotated and spliced three times to form a supercell, to derive the original structure of functionalized CNTs. Then, the density functional theory (DFT) was used to optimize the structure to derive the best geometry. The DFT calculation was conducted in Vienna ab initio simulation package (VASP) software package, where Perdew Burke ernzerhof (PBE) [43] was used as the exchange correlation function and a plane-wave cutoff energy was set to be 550 eV. The optimized structure was used to construct 2 × 2 carbon nanotube arrays, where the inter-tube distance was maintained at 0.6 nm. The simulation box containing CNT bundles has dimensions of 38 × 38 × 50 Å, and the periodic boundary conditions were applied in the x and y directions.

## 3. Results and Discussion

### 3.1. Effect of Pore Size on the Adsorption of CO_2_/N_2_ Mixture in CNTs

**Figure 2:**
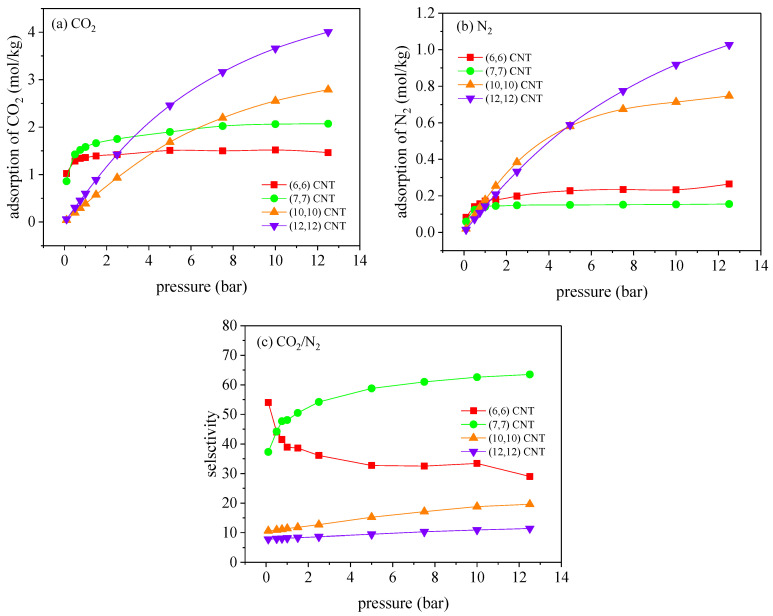
Adsorption isotherms of (**a**) CO_2_ and (**b**) N_2_, and (**c**) the variation of the corresponding CO_2_/N_2_ selectivity with pressure in different CNTs, at 300 K.

The adsorption of CO_2_/N_2_ mixture (with a mole ratio of 16/84 in the gas phase) in the CNTs at 300 K is conducted to derive the optimal diameter for CO_2_ capture, where the pore diameters varies from 0.81 to 1.63 nm. Figure 2 depicts the adsorption isotherms of CO_2_/N_2_ and the corresponding CO_2_/N_2_ selectivity at 300 K. As suggested, within the diameter range, all the adsorption isotherms of CO_2_ and N_2_ could be represented by type I according to the IUPAC classification. It is seen that the adsorption of CO_2_ and the CO_2_/N_2_ selectivity in the (6, 6) CNT with a diameter of 0.81 nm achieves their maxima below 1.0 bar. However, for the pressure range from 1.0 to 5.0 bar, the (7, 7) CNT with a diameter of 0.95 nm exhibits superior performance on separation CO_2_/N_2_ in comparison with the performance in the rest, in which both the adsorption of CO_2_ and the CO_2_/N_2_ selectivity are the highest. In the larger (10, 10) and (12, 12) CNTs, although the adsorption amount of CO_2_ monotonically increases with pressure, which is consistently higher than the result in the small CNT, the CO_2_/N_2_ selectivity is dramatically reduced compared with the value in the (6, 6) and (7, 7) CNTs. Consequently, the enlarged CNT diameter promotes the adsorption capacity of CO_2_ and N_2_ simultaneously, while reducing the CO_2_/N_2_ selectivity for the weak adsorbate–adsorbent interactions. Considering the superior adsorption amount of CO_2_ and significantly higher CO_2_/N_2_ selectivity, the (6, 6) and (7, 7) CNTs can provide great potential on CO_2_ separation from flue gas.

**Figure 3:**
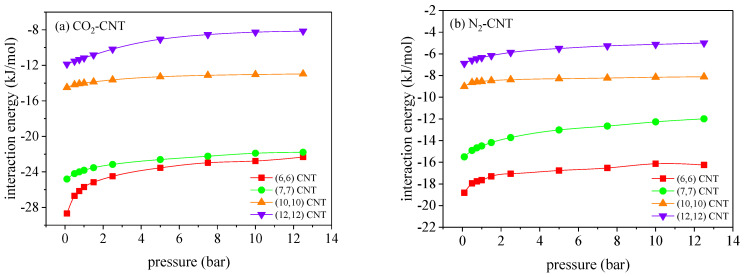
Variation of the interaction energies of (**a**) CO_2_-CNT and (**b**) N_2_-CNT with pressure, at 300 K.

It is understood that the adsorption of CO_2_/N_2_ mixture in the CNTs is determined by the competition effect between the adsorbate–adsorbent interactions and the entropic effect. Figure 3 illustrates the variation of CO_2_–CNT and N_2_–CNT interactions with pressures in the CNTs with different pore sizes, where the detailed calculating procedure was provided in our previous study [3]. Although both the CO_2_–CNT and N_2_–CNT interactions decrease with the pore size of CNTs, the dependency of interactions on the pore size is stronger for CO_2_. Accordingly, the preferential adsorption of CO_2_ over N_2_ is suppressed in the larger CNTs, leading to the reduced CO_2_/N_2_ selectivity. In consideration of the nominal diameter, $d_{CNT}$, of the (6, 6) CNT is 0.81 nm, the effective diameter for CO_2_ molecules rotating inside the (6, 6) CNT can be approximately measured as *d_eff_ = d_CNT_*−*σ_O−C_* = 0.49 nm, where $\sigma_{O-C}=0.32$ nm is determined according to (*σ_o_* *+* *σ_C_)/2*, using the LJ size parameters of carbon atoms ($\sigma_{C}$) of the CNT and oxygen atom ($\sigma_{O}$) of the CO_2_ molecule. As the molecule size of CO_2_ molecule (0.5331 nm) in the axial direction is larger and that for N_2_ molecule (0.441 nm), CO_2_ molecules in our simulations are found to distribute almost in parallel to the axis of the (6, 6) CNT, showing strong rotational restrictions. However, the rotational freedom of N_2_ is negligibly affected. In addition, random distributions of CO_2_ molecules are observed in the (7, 7) CNT with a diameter of 0.95 nm, suggesting that the dramatically enhanced entropic effect is responsible for the reduced CO_2_/N_2_ selectivity in the (6, 6) CNT, compared to the (7, 7) CNT.

### 3.2. Effect of Single Impurity on the Adsorption of CO_2_/N_2_ Mixtures in CNTs

**Figure 4:**
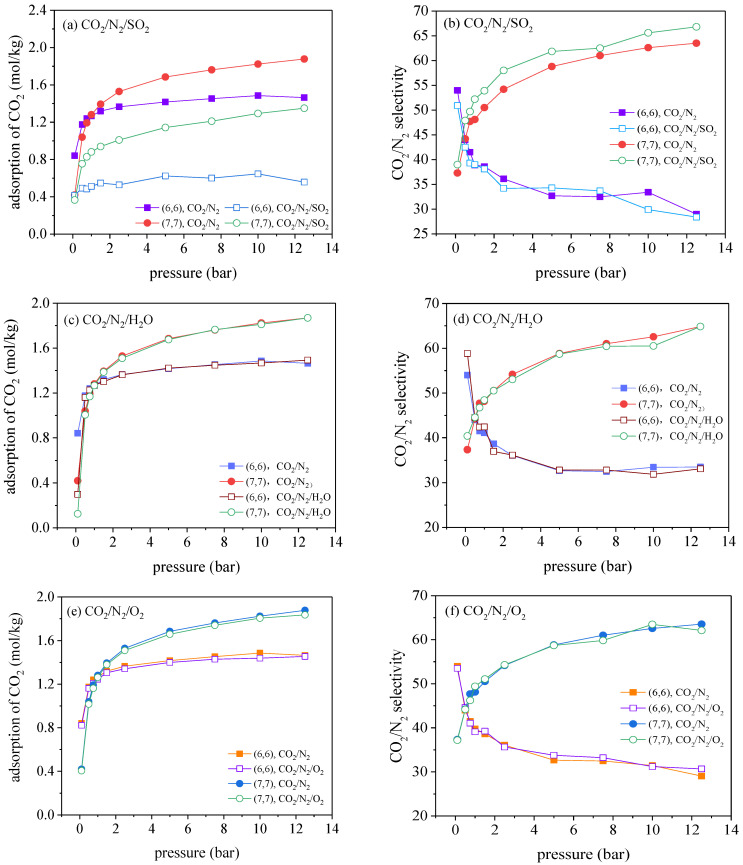
Adsorption isotherms for CO_2_ in the presence of impurities, (**a**) SO_2_, (**c**) H_2_O, and (**e**) O_2_, and (**b**,**d**,**f**) the corresponding CO_2_/N_2_ selectivity in the (6, 6) and (7, 7) CNTs, respectively.

The adsorption of ternary mixtures, CO_2_/N_2_/X in CNTs at 300 K, with X denoting a specific impure gas including H_2_O, SO_2_, and O_2_, is further investigated. It is found that insignificant impact of impurities on the separation of CO_2_ is found in the (10, 10) and (12, 12) CNTs in all the cases, so all the simulation results for the (10, 10) and (12, 12) CNTs are provided in Figure S1 in the Supporting Information, and the results for the (6, 6) and (7, 7) CNTs are explored. The results for the adsorption of CO_2_ and CO_2_/N_2_ selectivity in these two CNTs are plotted in Figure 4. The adsorption curves of three impurities are shown in Figure S2.

To quantify the inhibition effect of impurity gas on the adsorption of CO_2_, an inhibition coefficient is defined as

(3) $$I=\left(a_{b}-a_{im}\right)/a_{b}\times 100\%$$

where $a_{b}$ and $a_{im}$ represents the adsorbed amounts of CO_2_ for the binary CO_2_/N_2_ mixture and for the ternary CO_2_/N_2_/X mixture, respectively. As suggested, for the impure gas SO_2_, the inhibition coefficient in the (6, 6) CNT reaches up to 50.5%, 59.6%, and 61.9%, under the pressure of 0.1, 1.0, and 12.5 bar, respectively. Similarly, the inhibition coefficients in the (7, 7) CNT corresponds to 12.9%, 31.2%, and 28.1% under the same condition. However, as seen in Figure 4c, the impact of H_2_O on the adsorption of CO_2_ is significant at low pressure (0.1 bar), which yields an inhibition coefficient of 64.5%. When the pressure is increased to above 0.1 bar, the inhibition coefficient of H_2_O sharply reduces to be negligible. It is interesting to find that both the adsorption of CO_2_ and the CO_2_/N_2_ selectivity is barely affected by the presence of O_2_ in the gas phase.

**Figure 5:**
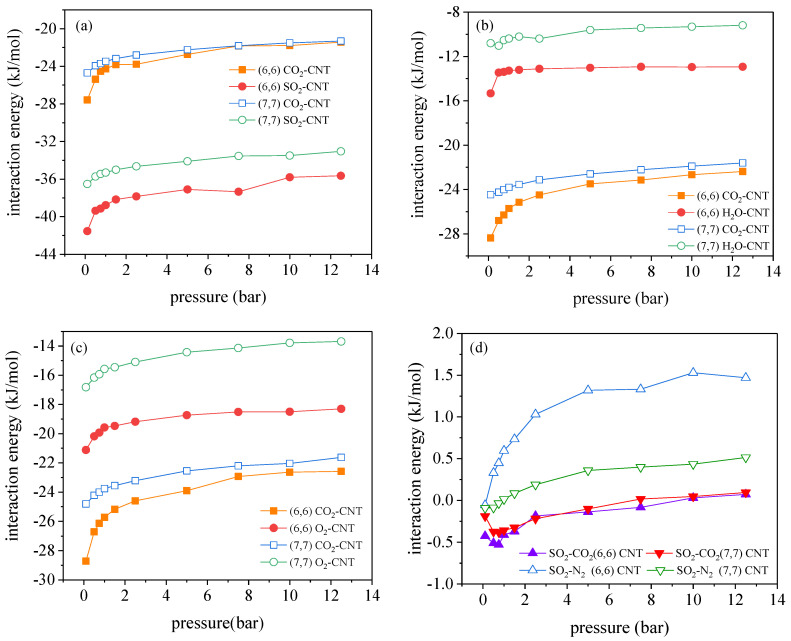
Variation of the interaction energies for CO_2_–CNT and impurity–CNT including (**a**) SO_2_–CNT, (**b**) H_2_O–CNT and (**c**) O_2_–CNT in the (6, 6) and (7, 7) CNTs with pressure. (**d**) The interaction energies of CO_2_–SO_2_ and N_2_–SO_2_ in the (6, 6) and (7, 7) CNTs for the CO_2_/N_2_/SO_2_ mixtures, determined from GCMC simulations at 300 K.

Figure 5a–c depicts the interactions of CO_2_–CNT and of impurity gas X-CNT in the (6, 6) and (7, 7) CNTs. As given in Figure 5, it is evident that SO_2_ has much stronger adsorption affinity with the nanotube wall than CO_2_, so strong adsorptive competition between SO_2_ and CO_2_ occurs, associated with the adsorption space being favorably occupied by SO_2_ molecules. Meanwhile, the interactions between CO_2_ molecules and the nanotube wall becomes weaker due to the introduction of SO_2_, so it is safe to conclude that the competitive adsorption and the weakened CO_2_–CNT interactions are responsible for negative impacts on the adsorption of CO_2_. Similar to the decreased adsorption of CO_2_, the adsorption of N_2_ also becomes smaller in the presence of SO_2_ (see Figure S3 in Supplementary Materials).

In addition, although both the adsorption amounts of CO_2_ and N_2_ are decreased by the presence of SO_2_ in the (6, 6) and (7, 7) CNTs, only a slight decrease in the CO_2_/N_2_ selectivity is found for (6, 6) CNT and the CO_2_/N_2_ selectivity is even enhanced in the (7, 7) CNT. To explain this phenomenon, the adsorbate–adsorbate interaction energies are estimated as a function of pressure for SO_2_–CO_2_ and SO_2_–N_2_ in Figure 5d. It is seen that CO_2_ molecules are strongly attracted by the adsorbed SO_2_ molecules in the (6, 6) and (7, 7) CNTs, whereas N_2_ molecules suffer the strong repulsions from SO_2_ molecules. As the additional CO_2_–SO_2_ interactions actually facilitate the selective adsorption of CO_2_ over N_2_, the CO_2_/N_2_ selectivity is enhanced by SO_2_ in the (7, 7) CNT. However, the adsorbed SO_2_ also enhances the entropic effect for CO_2_ adsorbing in the (6, 6) CNT, further restricting the rotation freedom of CO_2_ molecules, but this entropic effect exerts insignificant effect on the rotation of N_2_ molecules. Although the adsorption of CO_2_ is energetically favorable in the (6, 6) CNT in the presence of SO_2_, the strengthened entropic effect has completely dominated over the energetic effect, thereby leading to the dramatically reduced CO_2_ adsorption. The adsorption reduction arising from the dominant entropic effect is more significant for N_2_ due to its unfavorable energetic field exerted by SO_2_. Therefore, the CO_2_/N_2_ selectivity is reduced in the presence of SO_2_ in the (6, 6) CNT.

**Figure 6:**
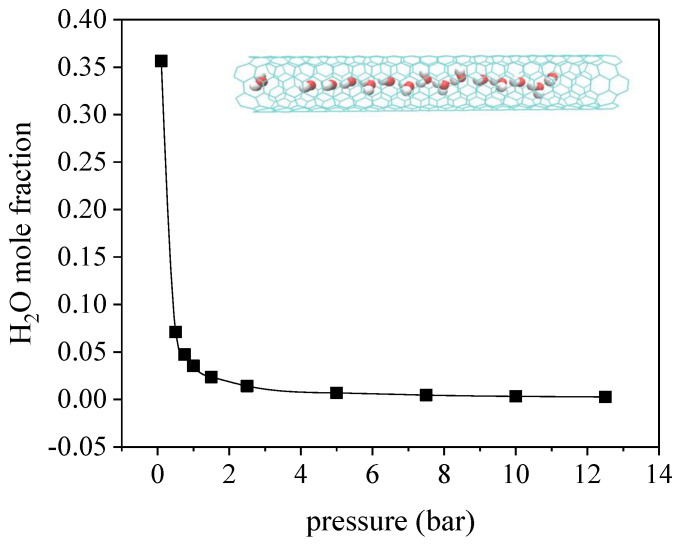
Variation of the H_2_O mole ratio with total pressure of CO_2_/N_2_/H_2_O mixture, with the partial pressure of H_2_O fixed at the saturation vapor pressure. The inset depicted a snapshot of the distribution of water in (6, 6) CNT at 0.1 bar and 300 K, in which a one-dimensional chain was evidently obtained.

Figure 4c indicates that, at the rather low pressure <0.1 bar (water vapor is at its saturation pressure, under a mole fraction of ~35.64%), noticeable adsorption of water vapor is found in the (6, 6) CNT, where considerable adsorption space is occupied. As depicted in Figure 6, the adsorption of water vapor decreases rapidly as a consequence of the competitive adsorption of CO_2_ and N_2_, where the corresponding partial pressures are enhanced at higher pressures. The inset of Figure 6 depicts the molecular configuration of water adsorbed in the (6, 6) CNT. As reported in the previous study, negligible adsorption of water was observed in the CNTs until the partial pressure of water vapor reached a critical pressure, where water molecules filled the CNT immediately and completely once the partial pressure was above the critical pressure [12,44]. It is shown that the critical pressure for the (6, 6) CNT is around the saturation pressure of water at 300 K, which is increased to 1.75 times of the saturation pressure in the (7, 7) CNT. Based on this reason, negligible adsorption of water is observed in the (7, 7) CNT within the pressure range investigated, while the effect of water vapor is only significant at rather low pressure in the (6, 6) CNT.

Figure 4e,f depicts the adsorption isotherms for CO_2_ and CO_2_/N_2_ selectivity in the presence of O_2_ in the (6, 6) and (7, 7) CNTs, where both the adsorbed amounts and the CO_2_/N_2_ selectivity are hardly affected. This result can be explained by the analysis of the interaction energy between guest molecules and CNTs. As given in Figure 5c, the interactions of O_2_–CNT are much stronger than the counterparts of N_2_–CNT, so the competitive adsorption occurs between O_2_ and N_2_, leading to an enhanced CO_2_/N_2_ selectivity. However, the concentration of O_2_ in the gas phase is only 4%, far below the mole concentration of N_2_, 84% of N_2_. Therefore, no significant decreases in adsorption of N_2_ occurred, which is also applicable to the result for CO_2_. A similar result is found in ZIF-68: the presence of O_2_ has a negligible effect on CO_2_ adsorption [12].

Apparently, the presence of impurity gas generally imposes a negative effect on the adsorption of CO_2_, particularly in the rather small CNTs. However, the CO_2_/N_2_ selectivity demonstrates a complex dependency on the impure gases, which can be enhanced, reduced, or nearly unaffected. Meanwhile, both the adsorption of CO_2_ and the CO_2_/N_2_ selectivity remain almost unaffected in the larger (10, 10) and (12, 12) CNTs, making it difficult to predict the optimal CNT with the highest separation performance. Therefore, it is necessary to introduce the performance coefficient, $\lambda_{e}$, which comprehensively evaluates the effect of the CO_2_ adsorption and the CO_2_/N_2_ selectivity on the separation performance, by following

(4) $$\lambda_{e}=\exp\left[\ln\left(\alpha_{1}\frac{M_{t}}{M_{p}}\right)+\ln\left(\alpha_{2}\frac{S_{t}}{S_{p}}\right)\right]$$

where $M_{t}$ and $S_{t}$ denote the adsorption of CO_2_ and the CO_2_/N_2_ selectivity for the CNT of interest at the target pressure, respectively, while $M_{p}$ and $S_{p}$ represent the adsorption of CO_2_ and the CO_2_/N_2_ selectivity for the standard case, respectively, which are chosen as the adsorption of CO_2_ and the CO_2_/N_2_ selectivity of the (7, 7) CNT at 300 K and 1.0 bar. $\alpha_{1}$ and $\alpha_{2}$ are the weight factor s, which are set as 1.0 in this work.

**Figure 7:**
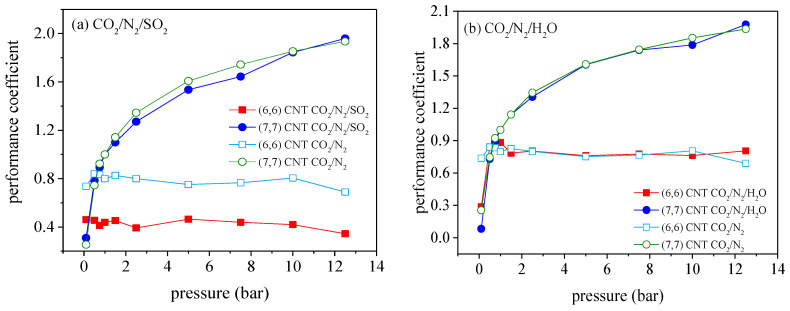

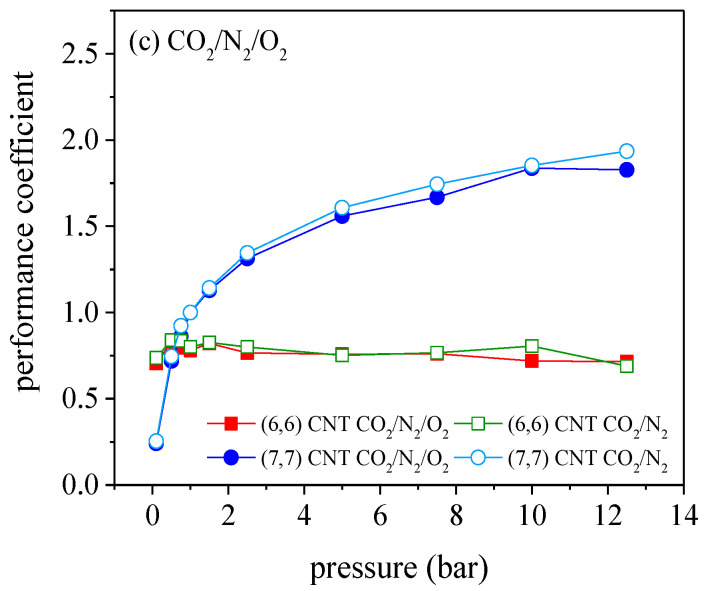
Variation of the performance coefficients of different CNTs in the presence of SO_2_ (**a**), H_2_O (**b**), and O_2_ (**c**), relative to the adsorption of binary CO_2_/N_2_ mixture (CO_2_/N_2_ is 16/84) in the (7, 7) CNT at 1.0 bar and 300 K.

Figure 7 illustrates the variation of the performance coefficient versus pressure in the (6, 6) CNT and (7, 7) CNT. As suggested, the performance coefficient is slightly increased in the (7, 7) CNT, while it becomes significantly decreased in the (6, 6) CNT. It is seen that SO_2_ exhibits the most influential impact on the adsorption of CO_2_ among the three impure gases considered. More specifically, the presence of SO_2_ dramatically reduces the performance coefficient in the (6, 6) CNT, which is 180% lower than the results for CO_2_/N_2_ mixture. This is caused by the strong competitive adsorption between SO_2_ and CO_2_. For the impurities of H_2_O and O_2_, the changes in performance coefficient are generally negligible, except for the results of CO_2_/N_2_/H_2_O mixture at 1 bar. Based on the above results, it is readily derived that the influence of impurities on the CO_2_ adsorption in CNTs followed the pattern: SO_2_ > H_2_O > O_2_. Figure 7 indicates that, in the presence of impurities, the (6, 6) CNT still provides better performances for CO_2_ capture than other CNTs when the pressures are below 0.5 bar, while the (7, 7) CNT exhibits the superior performance at higher pressures.

**Figure 8:**
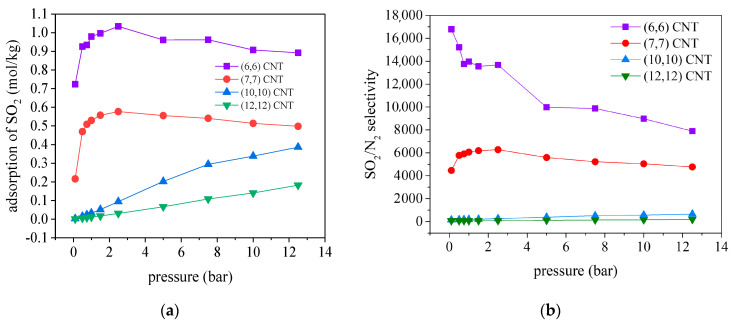
Adsorption of (**a**) SO_2_ and (**b**) SO_2_/N_2_ selectivity for the CO_2_/N_2_/SO_2_ in CNTs with diameter varied from 0.807 to 1.626 nm at 300 K.

Additionally, we explored the adsorptive separation performance of CNTs for capturing SO_2_ from the CO_2_/N_2_/SO_2_ mixture by measuring the isotherm curve of SO_2_ and the SO_2_/N_2_ selectivity, which are depicted in Figure 8. It should be pointed out that the (6, 6) CNT with a diameter of 0.81 nm exhibits outstanding performance for separation of SO_2_/N_2_, in which the maximum adsorbed amounts of SO_2_ and the highest selectivity are achieved among the CNTs considered. More specifically, the SO_2_/N_2_ selectivities are unprecedentedly high, reaching 16,796, 13,965 and 7892 at the pressures of 0.1, 1.0 and 12.5 bar at 300 K, in the (6, 6) CNT.

### 3.3. Impacts of Impurities on CO_2_ Capture in Functionalized CNT Arrays

From the previous simulation results, it is evident that SO_2_, as a polar molecule, yielded the strongest interaction with CNT, exerting the greatest impact on CO_2_/N_2_ adsorption and separation. As there are more complex interactions between impurities, it is interesting to explore the cooperative impact on the adsorption of CO_2_ in this part. Due to the hydrophobicity of carbon nanotubes, the adsorption of water molecules is weak, and a small amount of H_2_O barely affects the adsorption and separation of CO_2_/N_2_. In order to further explore the effect of H_2_O on CO_2_/N_2_ adsorption, the hydrophilic carboxyl modified CNT is studied. In order to keep the same number of carboxyl groups distributed on the unit cell of CNT with different diameters, the mass fraction of carboxyl group doping is about 5.01–9.64%. After structure optimization by DFT, a 2 × 2 carbon nanotube array is constructed. When the tube spacing is set at 0.6 nm, GCMC is used to simulate the gas adsorption in carbon nanotube arrays with different diameters, using a fixed temperature and gas composition. After simulation, the adsorption configurations inside and outside the carbon nanotubes are calculated, respectively.

**Figure 9:**
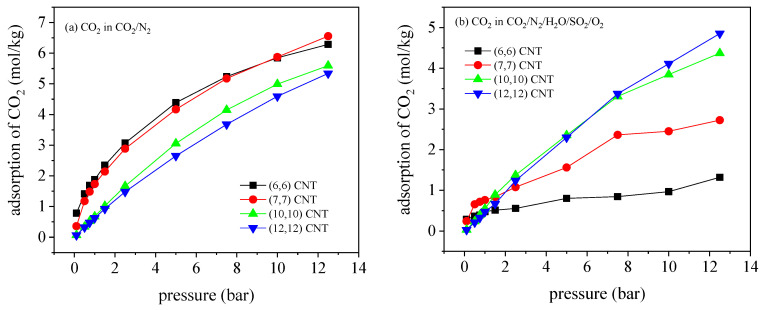

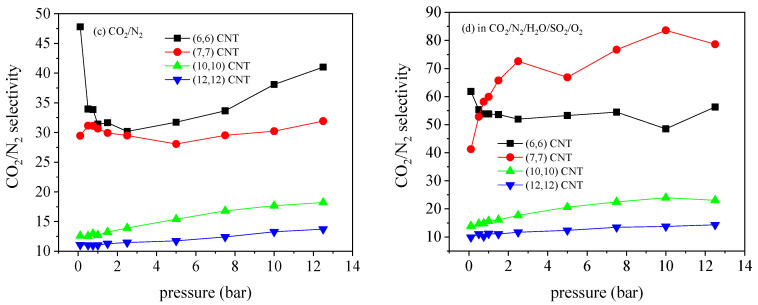
Adsorption isothermal curves of CO_2_ in (**a**) CO_2_/N_2_ mixture and (**b**) quinary mixture, as well as the corresponding CO_2_/N_2_ selectivities for (**c**) CO_2_/N_2_ mixture and (**d**) quinary mixture.

**Figure 10:**
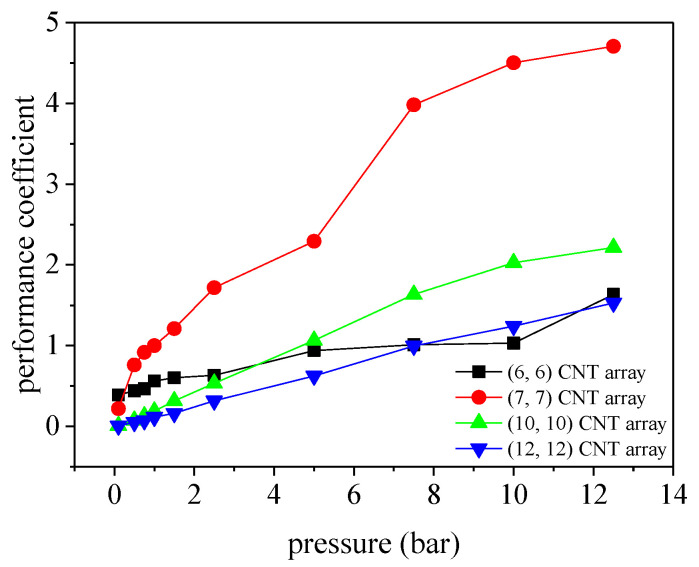
Performance coefficients of CO_2_/N_2_ adsorption and separation of quinary mixtures in modified CNTs with different diameters.

Figure 9 depicts the adsorption curves of CO_2_ and N_2_ and CO_2_/N_2_ selectivity mixed with impurity gases in four kinds of carbon nanotube arrays with tube spacing of 0.6 nm and temperature of 300 K. For CO_2_/N_2_ mixture, the optimal diameter of CNT bundle for adsorption separation of CO_2_ is found in the (6, 6) CNT, which is different from the result based on single CNT. This is because (6, 6) CNT not only has the strongest adsorbate CNT interaction, but also can provide additional adsorption space between tubes, so the adsorption capacity becomes enhanced. Under the combined effect of the two factors, (6, 6) CNT array has the best adsorption capacity and CO_2_/N_2_ selectivity under 10 bar. At higher pressure, due to the limited adsorption space, the adsorption capacity becomes lower than that for the (7, 7) CNT array. Compared with the binary mixture, the adsorption capacity of CO_2_ and N_2_ in quinary mixture is severely inhibited, especially in the small diameter (6, 6) CNT array, but the adsorption capacity of CO_2_ and N_2_ in the (7, 7) CNT array is the highest below 1 bar. In the (10, 10) and (12, 12) CNT arrays with large diameters, the adsorption capacity of CO_2_ increases almost linearly with the pressure, which becomes dominant when the pressure is greater than 1 bar. In addition, the CO_2_/N_2_ selectivity of the quinary mixture is increased. In particular, for (7, 7) CNT arrays, the adsorption capacity of CO_2_ and N_2_ decreased by 2.28 times and 4.45 times at 1 bar, respectively, but the selectivity increased by 1.95 times. This is because the inhibition effect is stronger for N_2_ (nonpolar molecule), in comparison with CO_2_. In addition, the selectivity of CO_2_/N_2_ in the quinary mixture is increased. By calculating the performance coefficient, as shown in Figure 10, it is found that (7, 7) CNT array always maintains the best adsorption separation performance, except some results at a very low pressure of 0.1 bar.

**Figure 11:**
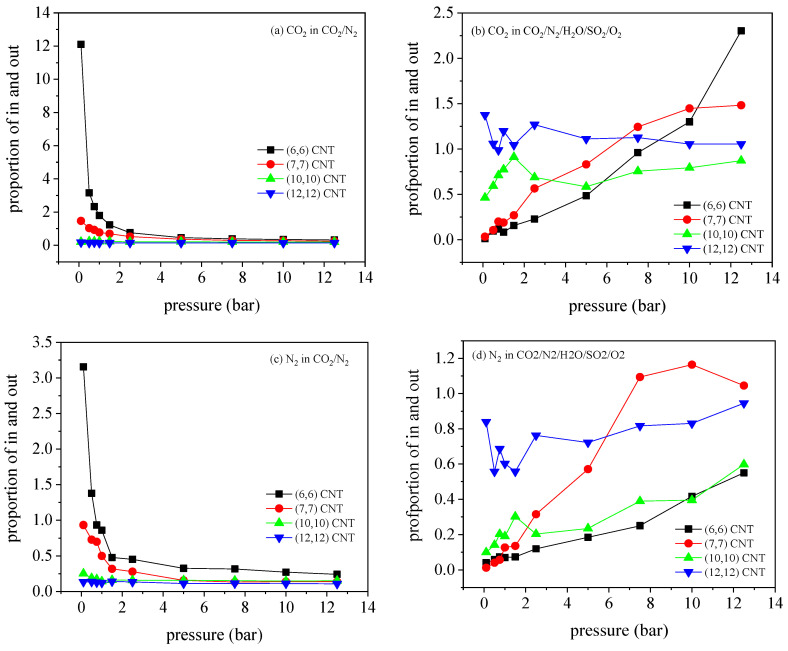
The ratio of adsorption capacity of (**a**,**b**) CO_2_ and (**c**,**d**) N_2_ in binary and quinary mixtures inside and outside the CNT arrays, with four different diameters.

In order to explore the inhibition mechanism in the CNT array with a small diameter, the adsorption ratio inside and outside the CNT (amount adsorbed inside the CNT/adsorption amount outside the CNT) is calculated. According to Figure 11 plotted the ratio of internal and external adsorption capacity for binary and quinary mixtures. As suggested, in the binary mixture, CO_2_ and N_2_ tend to be trapped by the outside of the tube in the small diameter, except some measurements at the pressure below 1 bar. This is due to the strong interaction between adsorbate and CNT in the small diameter below 1 bar. With increase in sorbate loading, the adsorption space in the tube is limited, so a large amount of adsorbate is captured by the outside of the tube. However, the interaction between adsorbate and CNT is weak in CNT with large diameter, so CO_2_ molecules tend to be adsorbed outside the tube. In the quinary mixture, the adsorption distribution of CO_2_ molecules is more complex. In the (12, 12) CNT array, CO_2_ molecules begin to be adsorbed mainly in the tube, which is distributed uniformly outside the tube with pressure. With the increase in the pressure, the pressure in the tube becomes dominant.

**Figure 12:**
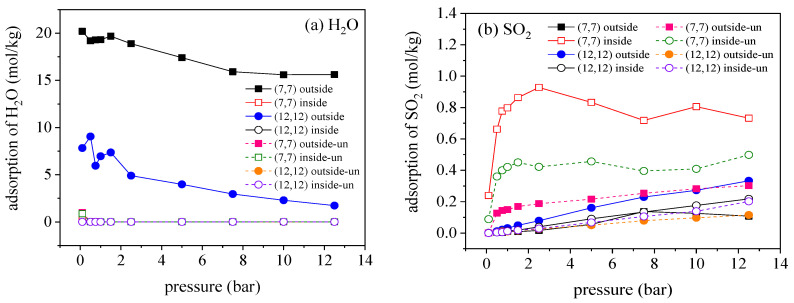
Isothermal adsorption curves of water molecules (**a**) and SO_2_ (**b**) inside and outside the tube in unmodified and modified CNT array.

**Figure 13:**
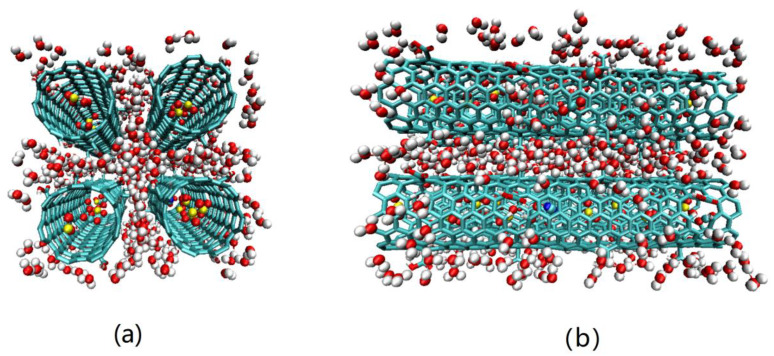
Molecular snapshot of (7, 7) CNT array in cross section (**a**) and axial direction (**b**) at 1.0 bar, 300 K.

**Figure 14:**
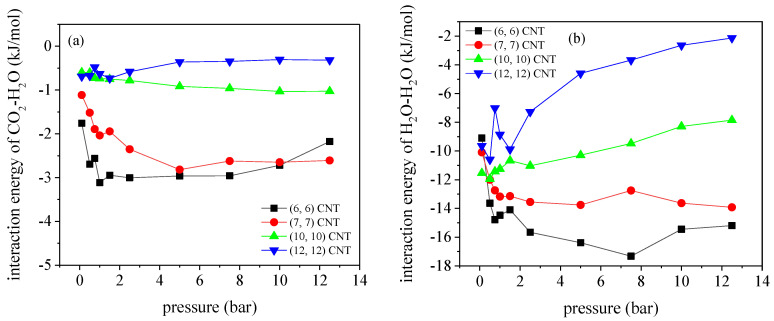

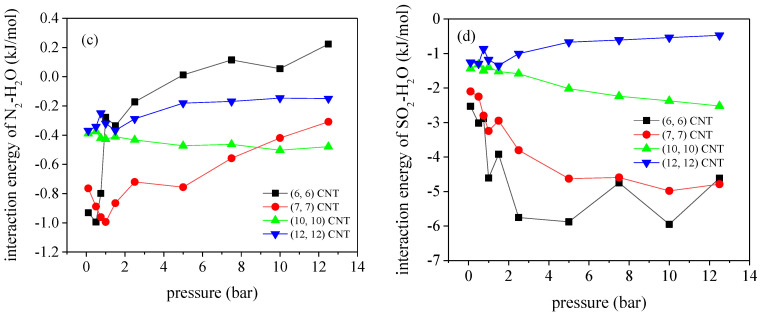
Interaction energy of CO_2_-H_2_O (**a**), H_2_O-H_2_O (**b**), N_2_-H_2_O (**c**) and H_2_O-CO_2_ (**d**) in CNT arrays with four diameters.

The isothermal curves of water molecules and SO_2_ in the modified CNTs are plotted in Figure 12, where the adsorption capacity of water molecules after carboxyl modification is greatly improved. The adsorption is mainly distributed between tubes, while the adsorption capacity inside tubes is almost zero. According to the molecular snapshot of water molecules adsorbed in (7, 7) CNT array in Figure 13, a large number of water molecules are adsorbed and aggregated between tubes to form chain structures, but the adsorption of water molecules in tubes is hardly observed. At the same time, the adsorption capacity of water molecules decreases with the increase in tube diameter. By calculating the mass fraction of doping carboxyl, it is found that the carboxyl content is an important factor to affect the adsorption capacity of water molecules. As the diameter of the tube increased, the mass fraction of carboxyl group decreases, leading to the decrease in the adsorption capacity of water molecules. The presence of water molecules promotes the adsorption of SO_2_ in the small-diameter nanotube arrays. In Figure 14, the results for interaction energy of H_2_O–SO_2_ indicate in the small-diameter (6, 6) and (7, 7) CNTs, SO_2_ is subject to stronger H_2_O–SO_2_ interaction than CO_2_–H_2_O, thereby enhancing the adsorption of SO_2_.

In the modified CNTs, carboxyl group has little effect on the adsorption of adsorbate molecules. By simulating the adsorption of quinary mixture in a single carboxyl modified CNT, the results show that the adsorption capacity of various adsorbents is reduced, in comparison with the simulation results for unmodified CNTs. This is due to the introduction of defect groups (or the lack of carbon atoms) which weaken the interaction between the adsorbate molecules and the wall of small-diameter CNTs, so the adsorption capacity is reduced. The introduction of carboxyl group barely promotes the adsorption and separation coefficient of adsorbate molecules in the carbon tubes, suggesting that H_2_O plays an important role in the adsorption capacity and distribution of CO_2_.

The adsorption of CO_2_ and N_2_ in the quinary mixture outside the tube is seriously inhibited, but the inhibition or promotion for adsorption inside the tube varies with nanotubes with different diameters. As the carboxyl functional group hardly exerts a positive effect on the adsorption of CO_2_ molecules in the tube, the adsorption of CO_2_ molecules in the tube is mainly affected by the interaction with other adsorbate molecules. Due to the large amount of adsorbed water molecules between the small nanotubes, the adsorption of CO_2_ molecules mainly occurs in the tube. However, at low pressures, the adsorption of SO_2_ in the tube is enhanced due to the presence of H_2_O. Meanwhile, the adsorption of CO_2_ in the tube is strongly inhibited by the intensive competitive adsorption, so CO_2_ adsorption mainly occurs outside the tube at low pressures. According to the previous simulation results of CO_2_/N_2_/SO_2_ mixture in a single CNT, SO_2_ has little effect on the adsorption of CO_2_ in a large diameter tube, so CO_2_ is mainly adsorbed in the tube at low pressure. With the increase in pressure, the adsorption amount of H_2_O outside the tube decreases, where the inhibition effect weakens, so CO_2_ molecules begin to adsorb outside the tube, and are finally evenly distributed inside and outside the tube. In addition, the adsorption enthalpy of CO_2_ is increased by the attraction of H_2_O–CO_2_ in the tube, where the adsorption space is abundant in the large diameter tube, so the adsorption of CO_2_ increases.

As derived from the previous analysis, SO_2_ can enhance the selectivity of CO_2_/N_2_ in the small diameter. In addition, CO_2_ is subject to stronger interaction from H_2_O than N_2_, so the presence of water can also promote the CO_2_/N_2_ selectivity. The selectivity of CO_2_/N_2_ in the small diameter is increased by the combination of the two impure gases. In particular, at 1 bar, the CO_2_/N_2_ selectivity of (6, 6) CNT array increases from 30.4 to 53.8, while an increase from 30.7 to 59.9 are found for (7, 7) CNT array. The growth ratio corresponds to 1.77 and 1.95 times, respectively. As the adsorption space in (6, 6) CNT array is very small, the derived adsorption capacity of CO_2_ is also very limited due to the competitive adsorption of H_2_O and SO_2_. For (7, 7) CNT array, the adsorption space is promoted, so the adsorption capacity of CO_2_ in (7, 7) CNT array becomes higher than that in (6, 6) CNTs. As the inhibition of CO_2_ in (7, 7) CNTs is weaker than that in (6, 6) CNT array, the selectivity of CO_2_/N_2_ is higher. To sum up, the adsorption of H_2_O molecules mainly occurs between tubes, thereby inhibiting the adsorption of CO_2_ between tubes, while SO_2_ molecules compete with CO_2_ molecules in tubes to induce the inhibition effect. The competition between the two effects determines the adsorption distribution of CO_2_ inside and outside the tube. In addition, the interaction of H_2_O and SO_2_ improves the selectivity of CO_2_/N_2_, and the (7, 7) CNT array maintains the best CO_2_/N_2_ adsorption and separation performance except the results at low pressure of 0.1 bar.

## 4. Conclusions

In this work, a grand canonical Monte Carlo simulation is used to investigate the influence of impurity gases, including water, SO_2_, and O_2_, on the adsorption of CO_2_ in singe CNTs and functionalized CNT bundles. Initially, the effect of pore size of CNT on the adsorption of CO_2_/N_2_ mixture is examined, and it is revealed that the adsorption capacity had a strong dependence on the CNT diameter. Further, the influence of single impure gas on the adsorption of CO_2_ in CNTs is explored. By calculating inhibition coefficient to evaluate the influence on the adsorption of CO_2_, results indicate that SO_2_ is the most influential impure to affect the adsorption of CO_2_/N_2_. By introducing SO_2_, the interaction of CO_2_-CNT became weaker. Meanwhile, SO_2_ could compete with CO_2_ for the adsorption site, which exerts a negative effect on the adsorption of CO_2_, so the adsorption amount of CO_2_ has a significant decrease. Furthermore, the (6, 6) CNT exhibits superior performance for adsorption separation of SO_2_/N_2_. As for H_2_O, due to the partial pressure decreases sharply with pressure, decrease on the adsorption of CO_2_ only occurs noticeably bellow 0.1 bar. The existence of O_2_ hardly changes the adsorption amounts andthe CO_2_/N_2_ selectivity. Moreover, the performance coefficient is calculated to evaluate the adsorptive separation of CO_2_ comprehensively. It is shown that SO_2_ was the most influential impure gas to affect the adsorptive separation of CO_2_ from flue mixture. Eventually, the coexisting influence of three impure gases is also investigated. The performance coefficient is also calculated for the complex correlation with the diameter; however, it is hardly affected by the complex interaction among adsorbates. Among our simulations, the (7, 7) CNT yields the superior performance for CO_2_ adsorption and separation, where both the maximum uptakes and the highest selectivity occurs to the ambient temperature and pressure.

## Figures and Tables

**Table 1:** Lennard–Jones parameters, partial charges, and configurational parameters of adsorbates and CNT [1].

Molecule	Site	LJ Parameters		Molecular Model			
		$\varepsilon /k_{B}\left(K\right)$	σ(nm)	X(nm)	Y(nm)	Z(nm)	Charge (e)
CNT	C	28.0	0.34				0.000
	C, H	35.220	0.355				−0.115
	C, RCOOH	52.840	0.375				0.520
	O(C), RCOOH	105.68	0.296				−0.440
	O(H),RCOOH	85.550	0.300				−0.530
	H, RCOOH	0.00015	0.000				0.450
	H, RC	100	0.242				0.115
CO_2_	C	27.0	0.280	0.0	0.0	0.0	0.70
	O	79.0	0.305	±0.1149	0.0	0.0	−0.35
N_2_	N	36.0	0.331	±0.055	0.0	0.0	−0.482
	COM	0	0	0.0	0.0	0.0	0.964
H_2_O	O	78.2	0.317	0.0	0.0	0.0	−0.848
	H	0	0	±0.0765	0.0	0.0587	0.424
O_2_	O	54.4	0.305	±0.0604	0.0	0.0	−0.112
	COM	0	0	0.0	0.0	0.0	0.224
SO_2_	O	57.4	0.301	±0.1235	0.0	0.0	−0.235
	S	145.9	0.362	0.0	0.0	0.0	0.471

## Data Availability

The data presented in this study are available on request from the corresponding authors.

## Supplementary Materials

The following supporting information can be downloaded online. Figure S1: Adsorption isotherms for CO_2_ in the presence of impurities, (a) SO_2_, (c) H_2_O, and (e) O_2_, and the corresponding CO_2_/N_2_ selectivity (b, d and f), in the (10, 10) and (12, 12) CNTs. Figure S2: Isotherm curves with pressure for (a) SO_2_ in CO_2_/N_2_/SO_2_, (b) H_2_O in CO_2_/N_2_/H_2_O, and (c) O_2_ in CO_2_/N_2_/O_2_, in (6, 6), (7, 7), (10, 10) and (12, 12) CNTs at temperature of 300 K. Figure S3: The adsorption of N_2_ in the presence of impurities which are (a, b) SO_2_, (c, d) H_2_O and (e, f) O_2_. The leaf side is these mixtures in the (6, 6) and (7, 7) CNTs, and the right side is that in (10, 10) and (12, 12) CNTs. Figure S4: Variation interaction energy of X-CNT which X represents SO_2_, H_2_O and O_2_ with pressure in the (10, 10) (a) and (12, 12) (b) CNTs.
