# Supplementary material for: Impact of Impure Gas on CO2 Capture from Flue Gas Using Carbon Nanotubes: A Molecular Simulation Study
Source: Molecules. 2022 Mar 1;27(5):1627. doi: 10.3390/molecules27051627 (PMC8912003; doi:10.3390/molecules27051627)
Supplement: Supplementary file 1 [file molecules-27-01627-s001.zip › molecules-1531177-supplementary.pdf]

molecules

Supplementary File

Impact of impurity gas on CO_2 capture from flue gas using carbon nanotubes: A molecular simulation study

Yiru Su[a,c], Siyao Liu[a,*] and Xuechao Gao[b,*]

a) Key laboratory of low-grade Energy Utilization Technologies and Systems, Ministry of Education, School of Energy and Power Engineering, Chongqing University, Chongqing 400044, China
b) State Key Laboratory of Materials-Oriented Chemical Engineering, College of Chemical Engineering, Nanjing Tech University, No. 30 Puzhu Road (S), Nanjing 211816, PR China
c) State Key Laboratory of Pollution Control and Resource Reuse, School of the Environment, Nanjing University, Nanjing 210023, China

1. Effect of single impurity on the adsorption of CO_2/N_2 mixtures in lager CNTs

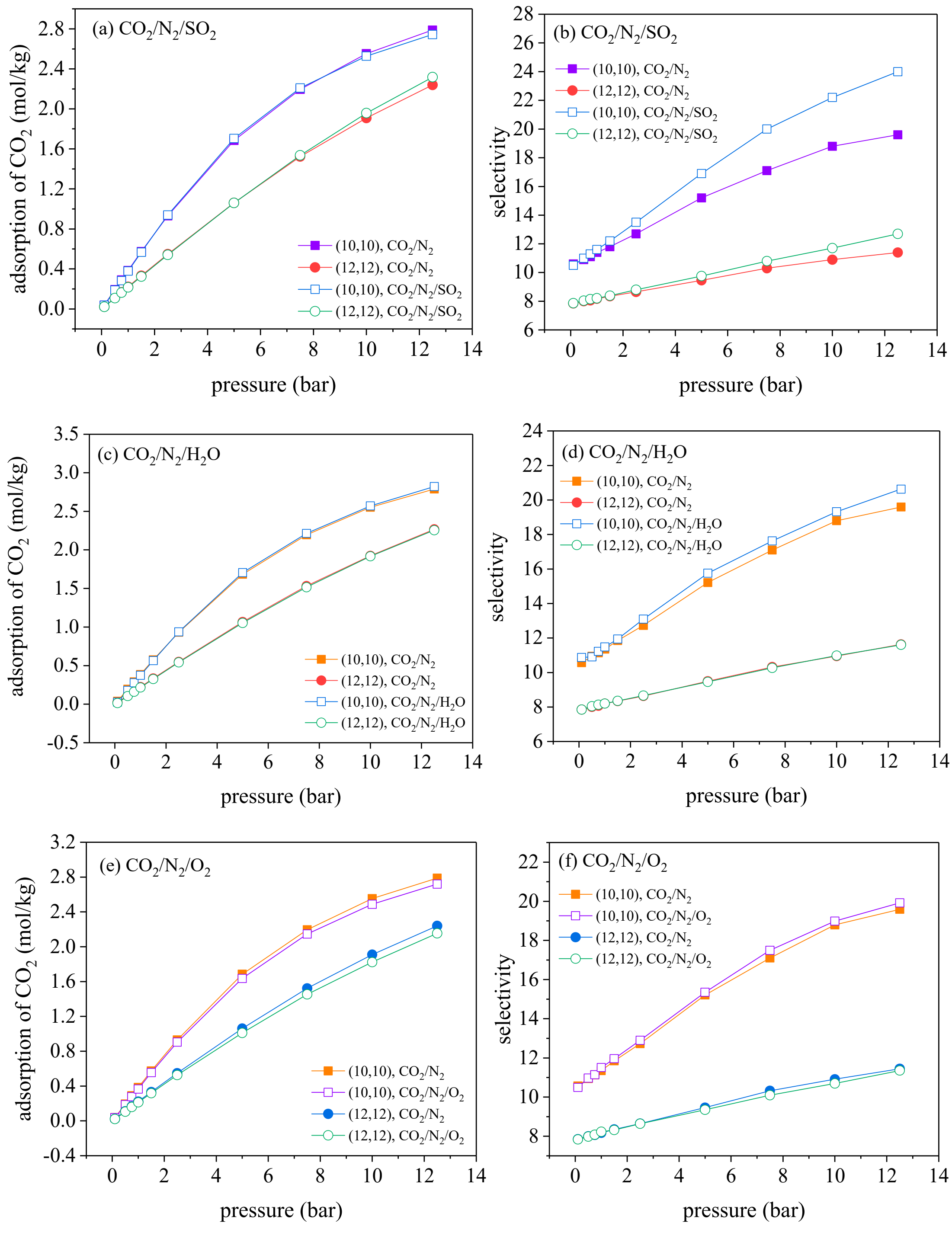

Figure S1. Adsorption isotherms for CO_2 in the presence of impurities, (a) SO_2, (c) H_2O, and (e) O_2, and the corresponding CO_2/N_2 selectivity (b, d and f), in the (10, 10) and (12, 12) CNTs.

2. Isotherm curves of single impurity in ternary mixtures

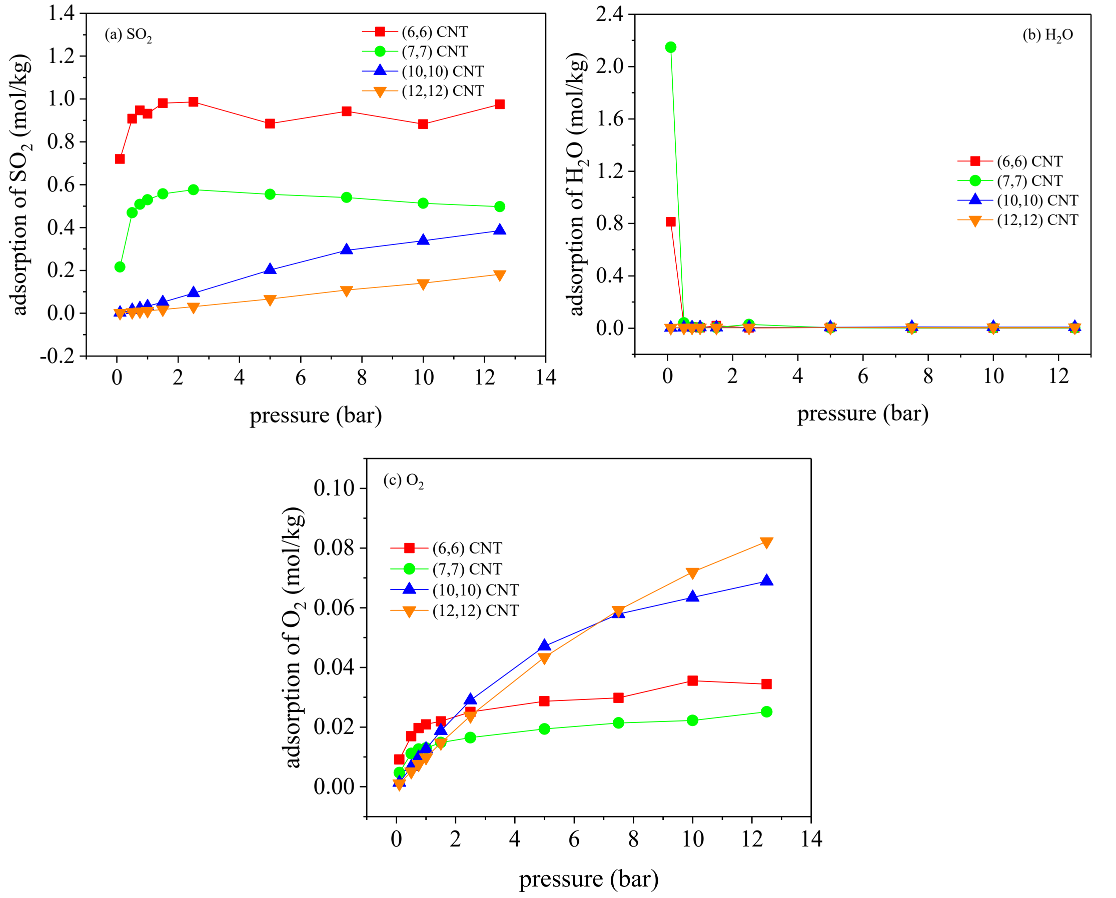

Figure S2. Isotherm curves with pressure for (a) SO_2 in $CO_2/N_2/SO_2$, (b) H_2O in $CO_2/N_2/H_2O$, and (c) O_2 in $CO_2/N_2/O_2$, in (6, 6), (7, 7), (10, 10) and (12, 12) CNTs at temperature of 300 K.

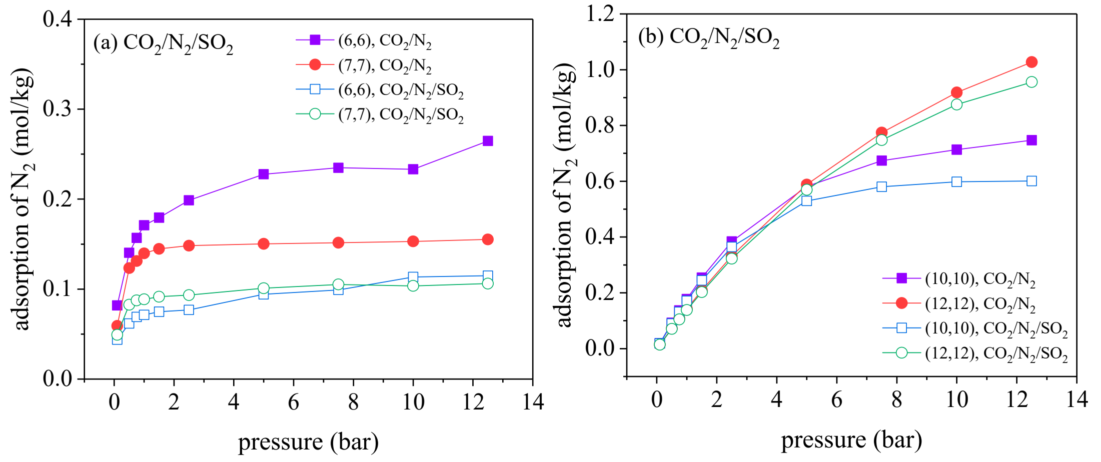

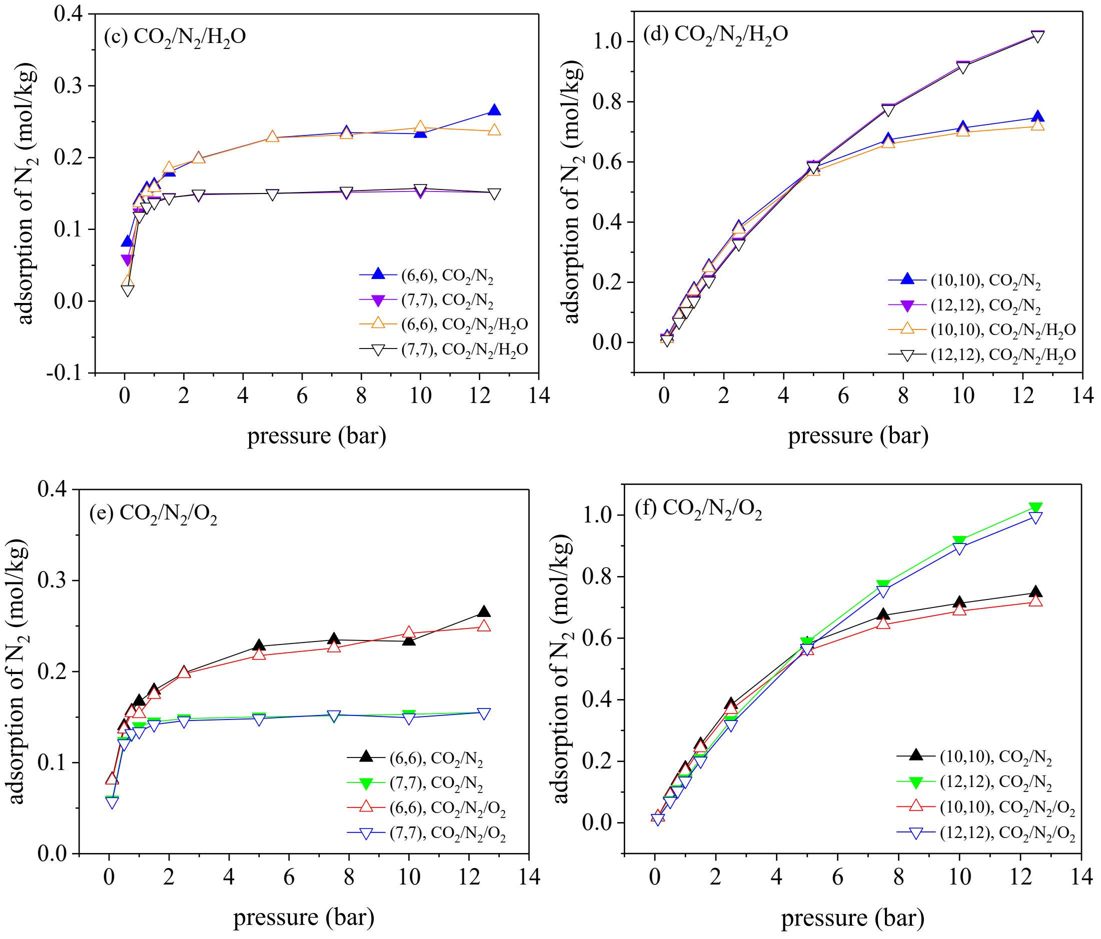

Figure S3. The adsorption of N_2 in the presence of impurities which are (a, b) SO_2, (c, d) H_2O and (e, f) O_2. The leaf side is these mixtures in the (6, 6) and (7, 7) CNTs, and the right side is that in (10, 10) and (12, 12) CNTs.

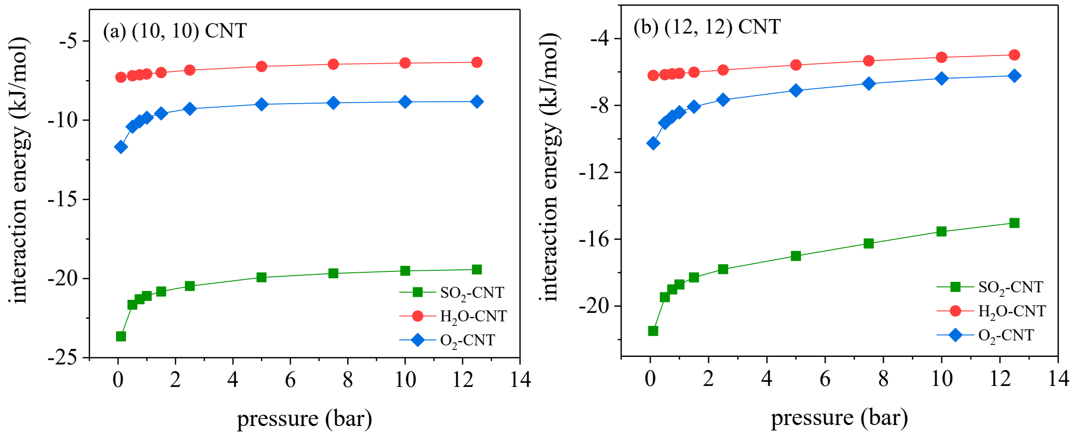

Figure S4. Variation interaction energy of X-CNT which X represents SO_2, H_2O and O_2 with pressure in the (10, 10) (a) and (12, 12) (b) CNTs.
